# Statins impact primary embryonic mouse neural stem cell survival, cell death, and fate through distinct mechanisms

**DOI:** 10.1371/journal.pone.0196387

**Published:** 2018-05-08

**Authors:** Ross A. Carson, Anthony C. Rudine, Serena J. Tally, Alexis L. Franks, Krystle A. Frahm, Jacob K. Waldman, Neerupma Silswal, Suban Burale, James V. Phan, Uma R. Chandran, A. Paula Monaghan, Donald B. DeFranco

**Affiliations:** 1 Department of Pharmacology and Chemical Biology, University of Pittsburgh School of Medicine, Pittsburgh, Pennsylvania, United States of America; 2 Department of Pediatrics, Division of Newborn Medicine, Children’s Hospital of Pittsburgh, University of Pittsburgh School of Medicine, Pittsburgh, Pennsylvania, United States of America; 3 Department of Pediatrics, Division of Child Neurology, Children’s Hospital of Pittsburgh, University of Pittsburgh School of Medicine, Pittsburgh, Pennsylvania, United States of America; 4 Department of Medicine, Division of Endocrinology and Metabolism, University of Pittsburgh School of Medicine, Pittsburgh, Pennsylvania, United States of America; 5 Department of Biomedical Informatics, University of Pittsburgh School of Medicine, Pittsburgh, Pennsylvania, United States of America; 6 Department of Biomedical Sciences, University of Missouri Kansas City School of Medicine, Kansas City, Missouri, United States of America; University of Manitoba, CANADA

## Abstract

Statins inhibit HMG-CoA reductase, the rate-limiting enzyme in the cholesterol biosynthesis pathway (CBP), and are used for the prevention of cardiovascular disease. The anti-inflammatory effects of statins may also provide therapeutic benefits and have led to their use in clinical trials for preeclampsia, a pregnancy-associated inflammatory condition, despite their current classification as category X (i.e. contraindicated during pregnancy). In the developing neocortex, products of the CBP play essential roles in proliferation and differentiation of neural stem-progenitor cells (NSPCs). To understand how statins could impact the developing brain, we studied effects of pravastatin and simvastatin on primary embryonic NSPC survival, proliferation, global transcription, and cell fate *in vitro*. We found that statins dose dependently decrease NSPC expansion by promoting cell death and autophagy of NSPCs progressing through the G1 phase of the cell cycle. Genome-wide transcriptome analysis demonstrates an increase in expression of CBP genes following pravastatin treatment, through activation of the SREBP2 transcription factor. Co-treatment with farnesyl pyrophosphate (FPP), a CBP metabolite downstream of HMG-CoA reductase, reduces SREBP2 activation and pravastatin-induced PARP cleavage. Finally, pravastatin and simvastatin differentially alter NSPC cell fate and mRNA expression during differentiation, through a non-CBP dependent pathway.

## Introduction

Statins are inhibitors of HMG-CoA reductase, the rate-limiting enzyme in the synthesis of cholesterol, and have widespread clinical use for the treatment of hyperlipidemia and prevention of cardiovascular disease. However, the therapeutic benefit of statins extends beyond the cardiovascular system [1,2], and results from preclinical models suggest that they may be neuroprotective in response to acute brain injury [3–5] and chronic neurodegenerative conditions [6–8].

Currently, prenatal statin administration is being evaluated for the prevention of preeclampsia [9] because of their potent anti-inflammatory effects on placental vasculature [10–13]. However, some clinical studies examining effects of statins *in utero* have called their safety into question leading to their designation as Category X drugs, meaning that they are completely contraindicated during pregnancy [14,15]. This was due to clinical reports of teratogenicity including limb and central nervous system (CNS) deformities [14], although more recent clinical [15] and animal studies [16] fail to replicate these findings. Given this uncertainty, statins are no longer excluded from clinical trials in pregnant women.

The concentration of cholesterol in the cerebral cortex increases during development [17], and ablation of squalene synthase (SQS), an enzyme in the *de novo* cholesterol biosynthetic pathway, results in failed embryonic neural tube closure [18]. In addition, conditional ablation of SQS in neural stem and progenitor cells (NSPCs) of the ventricular zone of embryonic mouse brain resulted in significant cortical atrophy due to death of newborn neurons [19].

Despite cholesterol’s essential role in brain development, beneficial effects of statins on the brain have been reported. In adult NSPCs, statins show potential in recovery from traumatic brain injury [3–5] and prevention of the progression of neurodegenerative diseases, such as Parkinson’s disease and Alzheimer’s disease [6–8]. Statins also have beneficial direct effects on NSPCs by increasing neurogenesis through enhancement of the Wnt/beta-catenin signaling pathway brought about by the inhibition of isoprenoid synthesis and influencing cell fate during differentiation [20]. Finally, maternal administration of pravastatin in a mouse model of preeclampsia reverses the reduced proliferation of NSPCs in offspring neocortex [21], as well as rescues vestibular function, balance and coordination test scores [22].

The potential for statins to provide benefits for disorders of pregnancy with limited therapeutic options warrants a more thorough understanding of its impact on fetal brain development. We therefore utilized primary mouse embryonic NSPC cultures to examine their specific molecular and cellular responses to both a hydrophobic (simvastatin) and hydrophilic (pravastatin) statin. The results of our studies demonstrate that statins induce cell death in NSPCs through inhibition of the cholesterol biosynthetic pathway (CBP) at high but not low doses. We also demonstrate that statins alter NSPC cell fate by a mechanism not dependent on CBP inhibition.

## Materials and methods

### Mouse cell culture

Timed-pregnant C57BL/6 mice were purchased from Charles River Laboratories (Raleigh, NC) and were housed individually and received chow and tap water *ad libitum*. All animal work was conducted according to relevant national and international guidelines. All animals were euthanized by inhaled CO_2_, 20–30% chamber volume exchange per minute. Animal work was approved by the University of Pittsburgh IACUC, PHS Assurance Number D16-00118 with Protocol Number 16037715. The entire cortical region was dissected from individual mouse embryonic day 14.5 embryos, dissociated into single cells, and grown as 3-dimensional neurosphere cultures in NeuroCult^TM^ media and proliferation supplement (STEMCELL 05702) as previously described [23,24]. Cultures were typically passaged every 5 days and experiments were performed between the third and fifth passage. NSPCs derived from male and female tissue was used for experiments, and fetal sex was determined by digesting tail tissue overnight at 56°C in proteinase K and using PCR analysis of isolated genomic DNA to detect the Y chromosome *Sry* gene.

### Cell growth assay

NSPCs were treated with vehicle, pravastatin (Sigma P4498) diluted in water and ethanol, or simvastatin (Sigma S6196) diluted in ethanol on the day after plating. Live images at 10x were analyzed using ImageJ. Counting was performed on dissociated NSPCs using a Nexelcom automated cellometer with trypan blue viability staining. Cell number on a given day was assessed for significance by two-way ANOVA and secondary Tukey test and doubling time analyzed by one-way ANOVA using Prism and is displayed as means with SEM.

### Flow cytometry

NSPCs were plated at 1.2x10^5^ cells/well in a Costar six-well ultra-low adherence plate and allowed to form neurospheres. Neurospheres were treated with vehicle (water and ethanol), pravastatin, or simvastatin and cultured for 3 days. Neurospheres were then collected by centrifugation, dissociated into single cells, washed in 1x phosphate buffered saline (PBS) (Corning 36415005) and fixed in 80% ethanol for 15 minutes on ice. They were then rehydrated and washed in PBS then stained in a 3 μM solution of propidium iodide (Sigma P4107), 100mM Tris Base (Fischer Scientific BP152), 150mM NaCl (VWR VW6430-5), MgCl_2_ (USB 18641) and 0.1% NP-40 (Calbiochem 492015). Stained cells were imaged on a Fortessa Flow System in the presence of propidium iodide and quantified using Diva software. Data were assessed for significance by two-way ANOVA and secondary Tukey test using Prism.

### Quantitative PCR

RNA was isolated from neurospheres using the Trizol extraction kit (Invitrogen 15596–026). cDNA synthesis was performed using the iScript Select cDNA synthesis kit (Bio-Rad; catalog no. 170–8897). Quantitative real-time PCR (qRT-PCR) was performed using a Stratagene Mx3000P with products generated using iTaq Universal SYBR Green Supermix (Bio-Rad; catalog no. 172–5121). Data were assessed for significance by either one-way ANOVA or two-way ANOVA and secondary Tukey test using Prism. Cells were treated with vehicle (water and ethanol), pravastatin, simvastatin, farnesyl pyrophosphate (Sigma F6892) diluted in ethanol, geranyl pyrophosphate (Sigma G6025) diluted in ethanol, or squalene (Sigma 3626) diluted in ethanol. Validated primers were selected from the Massachusetts General Hospital Primer Bank [25,26].

### Western blot analysis

NSPC protein lysates used in Western blot analysis were extracted in radioimmunoprecipitation assay buffer (10 mM Tris-Cl [pH 8.0], 1 mM EDTA, 0.5 mM EGTA, 140 mM NaCl, 1% Triton X-100, 0.1% sodium deoxycholate, 0.1% sodium dodecyl sulfate [SDS]) supplemented with 1x Halt Protease & Phosphatase Inhibitor Cocktail (Thermo Scientific #78446) and 1mM PMSF (Boehringer Mannheim 83659720). 15ug of protein/sample was separated on a SDS–7.5% PAGE gel and transferred to a polyvinylidene difluoride membrane (Millipore Immobilon-P IPVH00010). Western blot analysis was performed using SREBP2 (Abcam ab30682), cleaved PARP (Abcam ab30264), cyclin D1 (Sigma CC-7464), LC3B (Cell Signaling 2775), caspase 3 (Cell Signaling 9664), alpha tubulin (Cell Signaling 3873), beta actin (Santa Cruz 47778), and either anti-rabbit (Promega PR-W4001) or anti-mouse (Promega PR-W4021) horseradish peroxidase conjugated antibody. Bands were detected using the Western Bright ECL chemiluminescence detection system (Advansta K-12045-D50). Membranes were stripped and reprobed with the order of antibodies alternating. Images were quantified (densitometry) using ImageJ software. Data were assessed for significance by one-way ANOVA or two-way ANOVA and secondary Tukey test using Prism.

### Flow Cytometry to quantify EdU incorporation into proliferating cells

Cortical NSPCs were grown for three days then treated with pravastatin or simvastatin for three days. 4 hours prior to collection, 5- Ethynyl-2’-deoxyuridine (EdU) was added (10 μM). Cells were then harvested, manually dissociated into single cells, washed with ice cold PBS, and fixed with 4% paraformaldehyde. Fixed cells were processed using the Click-iT EdU Alexa Fluor 647 Imaging Kit (ThermoFisher Scientific C10340) according to manufacture instructions and EdU+ cells quantified using an LSRII flow cytometer (BD Biosciences).

### RNA-Seq library preparation

Cortical NSPCs were treated with 10 μM pravastatin or vehicle (PBS) for 24 hours and harvested in Trizol (Invitrogen 15596–026). An RNA-Seq library was prepared using the TruSeq Stranded Total RNA kit (Illumina RS-122-2201) and then subjected to next generation sequencing by the Tufts University Genomics Core.

### RNA-Seq data analysis

After sequencing, the reads were assessed for quality using the FastQC tool. Technical replicates were merged and then aligned to the Ensembl GRCm38 Reference Mouse genome using Tophat2 v2.1.1. Transcript assembly was performed using Cufflinks v2.2.1 and the resulting Cufflinks assemblies from each sample were merged with the reference GTF file using Cuffmerge. Expression was quantified using HTSeq with the merged GTF file and default parameters. EdgeR was used to find the genes differentially expressed between different conditions and further refined to only contain genes up- or down-regulated by pravastatin by greater than 1.5-fold. Differentially expressed genes from each of the comparisons were uploaded to Ingenuity Pathway Analysis for functional analysis (version 1.0; QIAGEN). RNA-Seq data have been deposited at GEO with GSE identification number GSE111945.

### NSPC differentiation

NSPCs were cultured as adherent cells plated at a density of 2.4x10^5^ cells/well in a 6-well cell plate (Costar 3561) or 1.2x10^5^ in a 24-well plate on glass coverslips (Costar 3524) sequentially pre-treated with poly-D-lysine and laminin. Adherent NSPCs grown in NeuroCult media and proliferation supplement as previously described [23,27] were grown to 70–80% confluency. Proliferation media was then replaced with NeuroCult media and differentiation supplement (STEMCELL 05704). Differentiating cells were grown in the presence of 10 μM pravastatin, 1 μM simvastatin, or vehicle for three or four days before collection for mRNA or immunostaining. qRT-PCR was analyzed with one-way ANOVA with a secondary Tukey or Sidak test in Prism.

### Immunostaining and imaging

NSPCs were fixed in freshly prepared 4% paraformaldehyde pH 7.4, washed consecutively with PBS, then PBS plus Tween 20 (PBST), and finally PBS for 5 minutes each. Fixed and washed cells were then treated for 30–45 minutes with 5% heat inactivated normal goat serum and then incubated with the primary antibody overnight at 4°C. Primary antibodies included anti-2'3'-cyclic nucleotide 3'-phosphodiesterase (CNPase) (Abcam ab6319) to visualize oligodendrocytes, anti-beta-3 tubulin (Abcam ab 107216) to visualize neurons and anti-glial fibrillary acidic protein (GFAP) (Thermo Fisher PA3 16727) to visualize astrocytes. Cells were washed 3 times each for 5 min each in PBS, then PBST and finally PBS prior to incubation with secondary antibodies for 40 minutes at room temperature. Secondary antibodies included goat anti-mouse Cy3 (Abcam ab6939), goat anti-chicken Alexa Fluor 647 (Abcam ab150171), and goat anti-rabbit Alexa Fluor 488 (Abcam ab150077). Cells were then washed 3 times each for 5 min each in PBS, then PBST and finally PBS with the inclusion of DAPI in the final PBS wash. Fluorescent images were captured on an Evos 2 station microscope at 20X. The number of DAPI and GFAP, CNPase and beta-3 tubulin cells were counted and assessed for statistically significant differences using ANOVA with secondary Tukey test in Prism software.

## Results

### Statins decrease NSPC expansion

**Fig 1:**
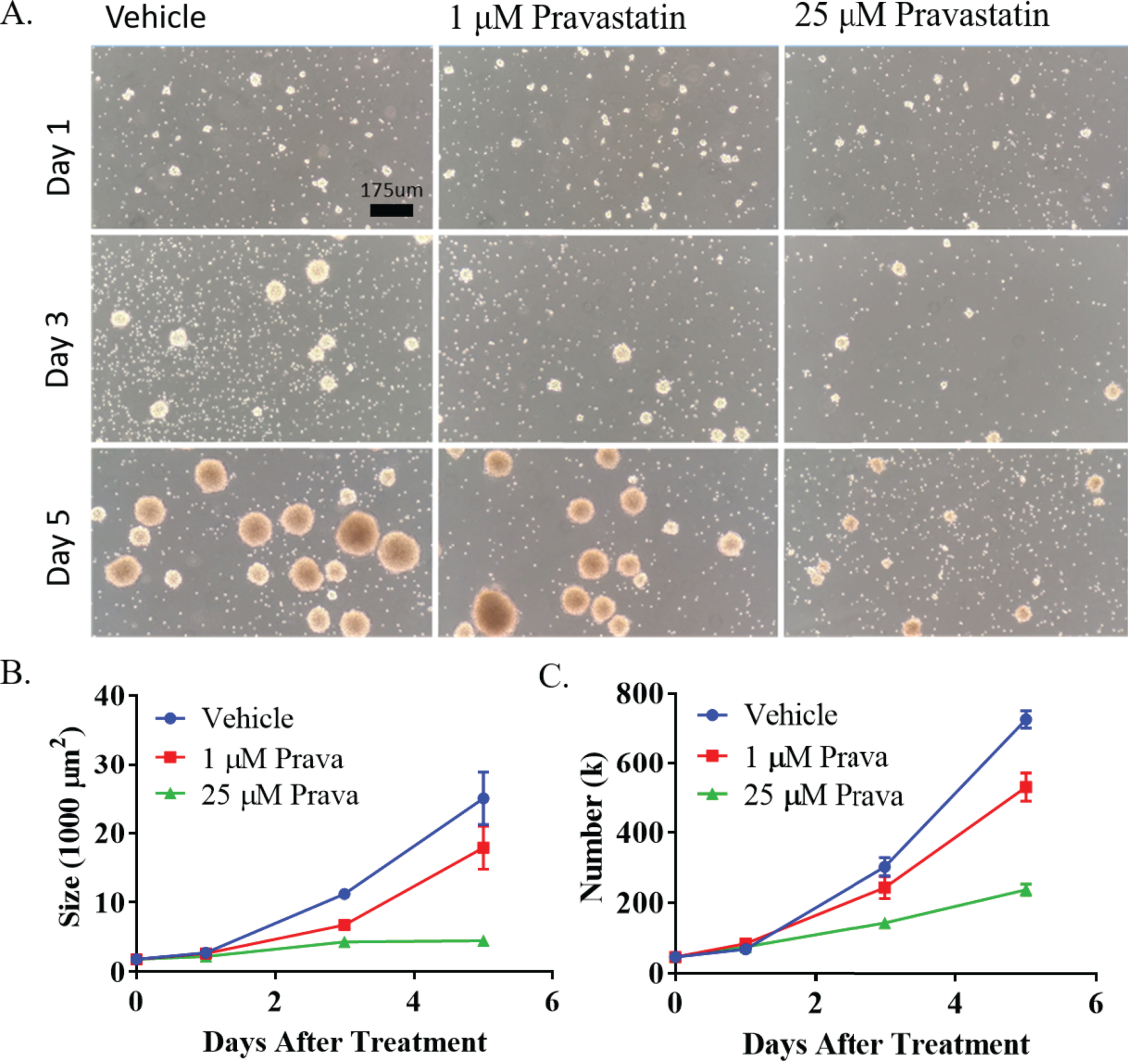
Pravastatin decreases NSPC expansion *in vitro*. (A) Representative images of neurosphere cultures. Treatments include vehicle, 1 μM and 25 μM pravastatin (prava) on days 1, 3 and 5 after treatment. Bright field images were taken on an inverted microscope at 10x magnification. (B) Quantification was performed on images taken in triplicate using image J software on days 0, 1, 3 and 5 after treatment with vehicle, 1 μM or 25 μM prava (n = 5). (C) After imaging, NSPCs were collected for automated cell counting in duplicate (n = 5).

Dissociated NSPCs were grown as neurospheres in the presence of vehicle, low, or high doses of pravastatin (1 μM and 25 μM) or simvastatin (0.1 μM and 5 μM). To determine effects on NSPC viability, neurosphere size was assessed by live imaging and cell viability performed by automated cell counting at one, three and five days after treatment. Vehicle treated NSPCs formed large neurospheres with an average cross sectional area of 25,000 μm^2^ five days after treatment, while NSPCs treated with low or high dose pravastatin reached average cross sectional areas of only 17,000 μm^2^ (p<0.001) and 4,500 μm^2^ (p<0.0001) respectively (Fig 1A and 1B). NSPCs treated with low or high doses simvastatin demonstrated similar effects on neurosphere size (S1A Fig). Therefore, both hydrophobic and hydrophilic statins limit the expansion of stem or progenitor cells present in primary neurosphere cultures.

To provide independent validation of stem and progenitor cell proliferation, effects of statins on the expansion of individual cells were determined. Vehicle treated NSPCs reached an average number of 724,500 cells/well five days after treatment, while NSPCs treated with low or high dose pravastatin only 531,500 cells/well (p<0.0001) and 239,300 cells/well (p<0.0001) respectively (Fig 1C). NSPCs treated with low or high dose simvastatin exerted similar effects on cell number (S1B Fig).

#### Statins induce cell death in NSPCs

**Fig 2:**
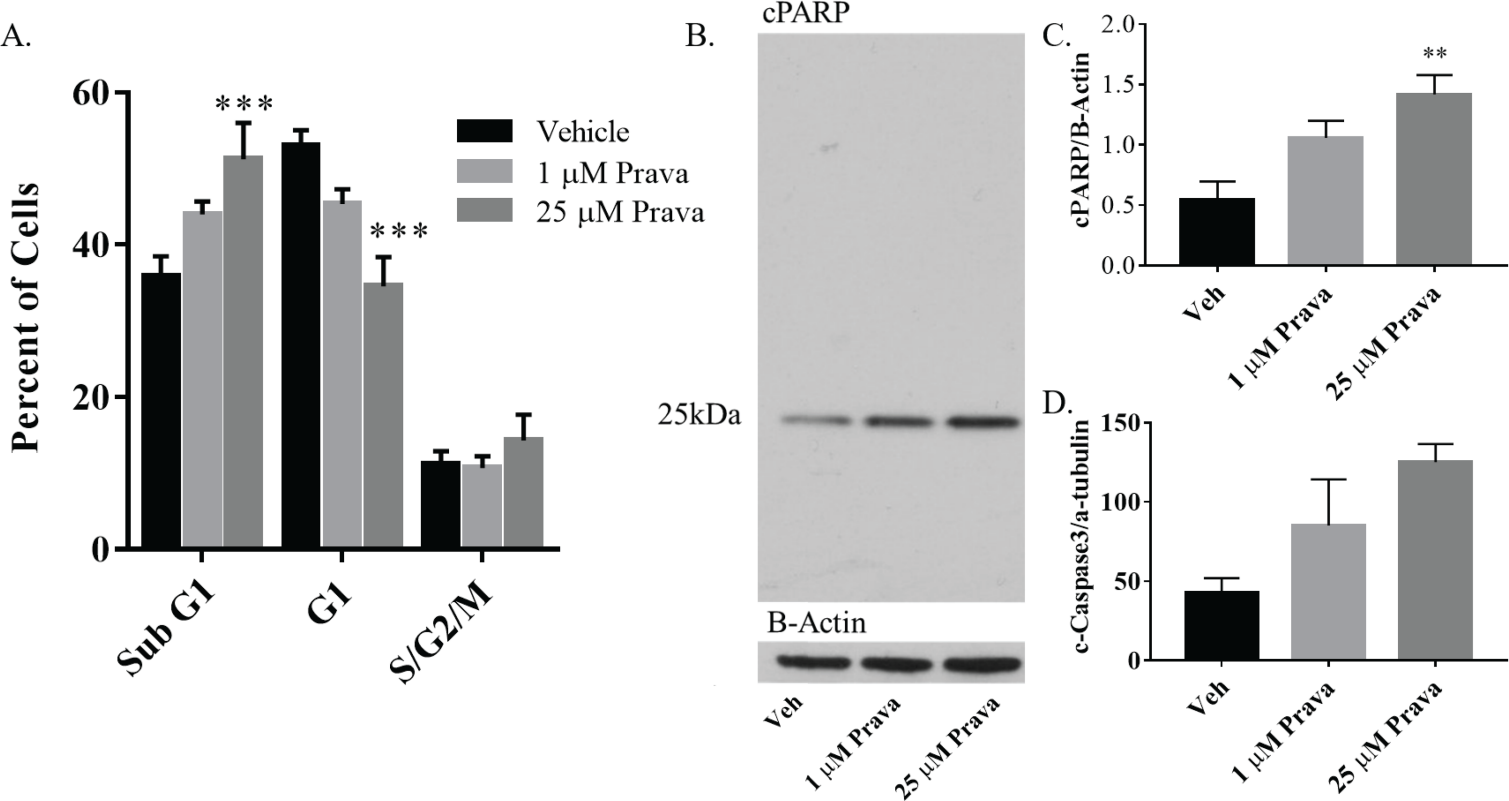
Pravastatin enhances cell death and reduces G1 accumulation of NSPCs. A. Quantification of NSPCs in sub-G1, G0/G1 and S/G2/M. Diva software was used to count the number of NSPCs that were identified as sub-G1, in G0/G1 or in S/G2/M phase. The histogram represents the mean percentage of NSPCs and standard error of the mean (SEM) in each category after treatment with vehicle, 1 uM or 25 uM prava (n = 5). B. Representative western blot of anti-cleaved PARP (cPARP) from neurospheres treated with vehicle, 1 uM or 25 uM pravastatin (prava) for 24 hours. C. Average intensity of bands corresponding to 24 hour treatment with vehicle, 1 uM or 25 uM prava for cleaved PARP normalized to ß-Actin (n = 5). D. Average intensity of bands corresponding to 24 hour treatment with vehicle, 1 uM or 25 uM prava for cleaved caspase normalized to alpha-tubulin (n = 3). (**p<0.01, ***p<0.001, compared to vehicle).

To determine whether decreased cell cycle progression or increased cell death was responsible for reduced NSPC expansion after statin treatment, neurospheres were treated with low and high doses of pravastatin or simvastatin for three days. They were then collected, fixed and stained with propidium iodide for analysis by flow cytometry. Gating for phases of the cell cycle was set by NSPCs arrested in G1 to detect NSPCs in G1, S/G2/M, and sub-G1. NSPCs demonstrated a dose dependent increase in sub-G1 cells in response to pravastatin treatment, which was statistically significant after high (p<0.001) but not low dose pravastatin treatment (Fig 2A). NSPCs treated with simvastatin demonstrated a similar effect on the accumulation of sub-G1 cells (S2A Fig). To determine the contribution of apoptosis to the accumulation of sub-G1 cells, cleavage of PARP (cPARP) and caspase 3 were examined. Western blot analysis revealed a statistically significant, dose dependent increase in PARP and cleavage and a trend towards caspase 3 cleavage in NSPCs treated pravastatin (Fig 2B–2D). The small amount of cPARP seen in vehicle treated cells is variable and may represent dead cells that accumulate in the center of neurospheres. NSPCs treated with simvastatin demonstrated similar effects on PARP cleavage after treatment (S2B Fig). Therefore, increased apoptosis or activation of other death inducing PARP cleavage proteases contributes to reduced NSPC expansion triggered by statin treatment.

#### Statins induce expression of cyclin D1, but inhibit cell cycle progression

**Fig 3:**
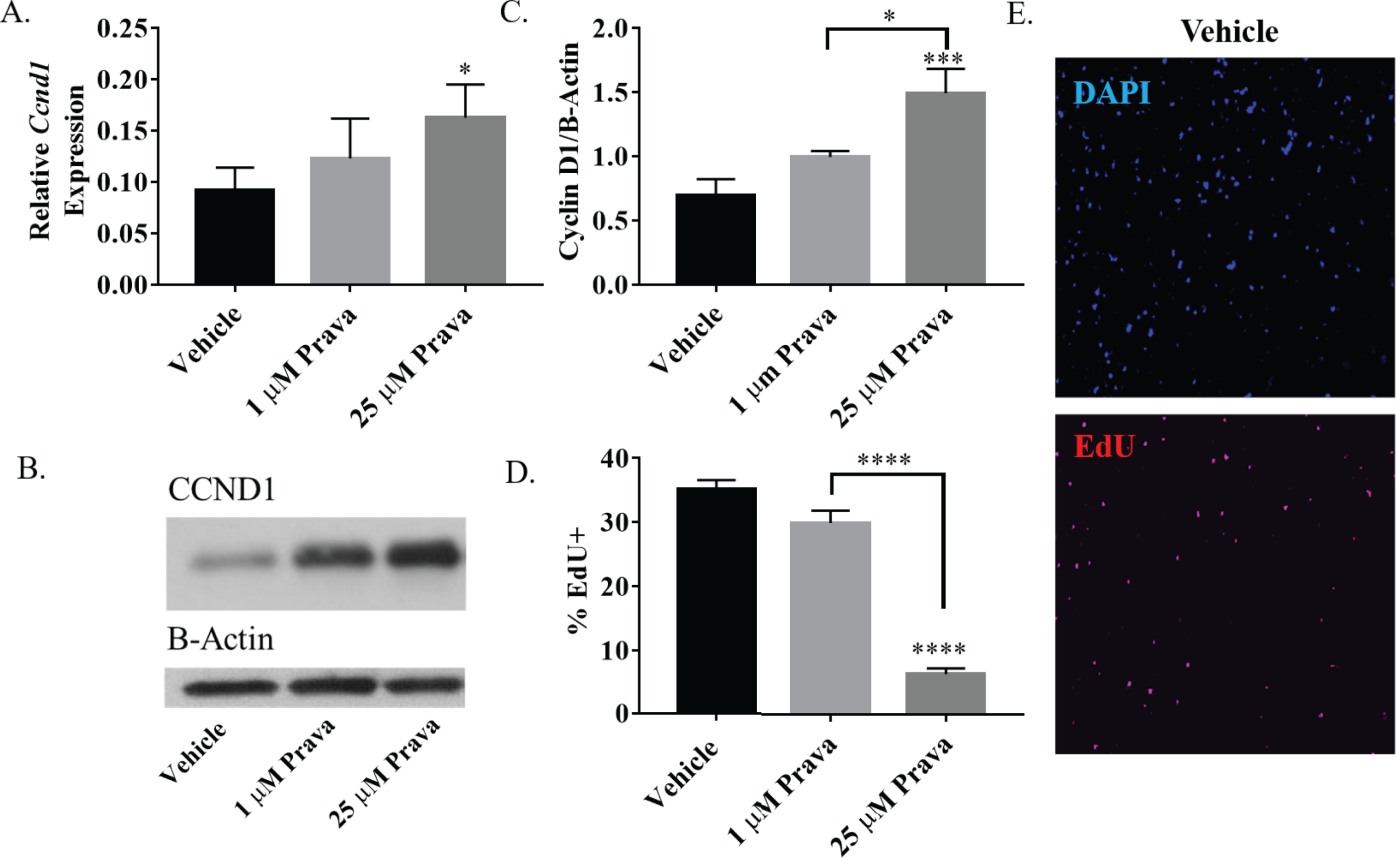
Pravastatin increases cyclin D1 expression but prevents passage through S-Phase in NSPCs. A. Average expression of *Ccnd1* mRNA as assessed by qRT-PCR (n = 5). B. Representative western blot of cyclin D1 from neurospheres treated with vehicle, 1 uM or 25 uM prava for 24 hours C. Average intensity of bands corresponding to 24 hour treatment with vehicle, 1 uM or 25 uM prava for cyclin D1 normalized to ß-Actin (n = 5). D. Percentage of EdU+ NSPCs treated with 1 uM or 25 uM prava for three days with EdU incorporation during the final 4 hours, quantified by flow cytometry (n = 8). E. Representative image of dissociated neurospheres after three days treatment with vehicle for DAPI and EdU incorporation. (*p<0.05, **p<0.01, ***p<0.001, ****p<0.0001 compared to vehicle, unless otherwise indicated by a horizontal bracket).

Flow cytometry analysis also revealed a dose dependent decrease of cell cycle progression of NSPCs after treatment with pravastatin and simvastatin. Specifically, the percentage of NSPCs in the G1 phase of the cell cycle was reduced by high (p<0.001), but not low dose pravastatin treatment (Fig 2A). NSPCs treated with simvastatin demonstrate similar trends (S2A Fig), suggesting that statins either cause an increase in cell cycle progression between G1 and S phases or increase apoptosis selectively in cells progressing through G1. We therefore examined expression of cyclin D1, a critical regulator of progression from G1 to S phase, after statin treatment. RNA and protein were collected for quantitative analysis from neurospheres treated for 24 hours with low or high dose pravastatin and simvastatin. qRT-PCR revealed a dose dependent increase in *cyclin D1* mRNA after pravastatin treatment, which demonstrated statistical significance at high (p<0.05) but not low dose pravastatin treatment (Fig 3A). Western blot analysis also demonstrated the same response, with a significant increase in the relative amount of cyclin D1 protein at high dose (p<0.001) but not low dose pravastatin treatment (Fig 3B and 3C). NSPCs treated with simvastatin demonstrate similar effects on *cyclin D1* mRNA and protein (S2C and S2D Fig). In summary, there appears to be a paradoxical increase in the cyclin that drives progression from G1 to S phase of the cell cycle (i.e. cyclin D1) in NSPCs associated with cell death.

Because statins do not cause a significant increase in the fraction of NSPCs in S/G2/M (Figs 2A and S2A), we examined NSPCs progressing through S phase using an EdU incorporation assay. NSPCs were treated with dose pravastatin for three days and then exposed to a 4 hour pulse of 10 μM EdU before collection. Interestingly, high (p<0.001) but not low dose pravastatin treatment caused a significant decrease in EdU incorporation (Fig 3D and 3E). Therefore, statins dose dependently reduce the progression of cells through S phase of the cell cycle and trigger cell death, predominately of cells trapped in G1.

#### Statins increase autophagy in NSPCs

In addition to apoptosis, we explored whether statins may also change autophagic activity in NSPCs as decreases in cholesterol are known to increase autophagic activity in CNS cells [28]. Cells were treated with low or high dose pravastatin for 24 hours and protein lysates were collected to quantify LC3B-II/(LC3B-I+LC3B-II) ratio, a sensitive indicator of autophagic activation. NSPCs treated with pravastatin demonstrated a modest, but significant (p<0.01) increase in autophagy when compared to both vehicle treated control and low dose pravastatin (S3A and S3B Fig).

#### Statin-regulated transcriptome in NSPCs

To determine whether transcriptional changes induced by statin treatment of NSPCs impact apoptosis and cell cycle dysregulation, the genome-wide transcriptome was analyzed following pravastatin treatment of NSPCs using RNA sequencing (RNA-Seq) technology. NSPCs derived from three female and three male embryos were treated with 10 μM pravastatin for a total of 24 hours and mRNA was collected for RNA-Seq analysis. Transcripts were identified as significantly regulated if they had a false discovery rate (FDR) of less than 0.05, greater than 1.5-fold change between treatment groups, and greater than one count per million transcripts. In NSPCs derived from male and female embryonic cortex, whole transcriptome sequencing identified 16,108 unique mRNA transcripts. While over 200 transcripts were significantly upregulated only two transcripts (potassium voltage-gated channel subfamily J member 4 and glutathione synthase) were significantly downregulated. The top 50 regulated transcripts sorted by p-value can be found in Supplemental Table 1 (S1 Table).

#### Induction of cholesterol biosynthetic pathway genes by statins in NSPCs

**Fig 4:**
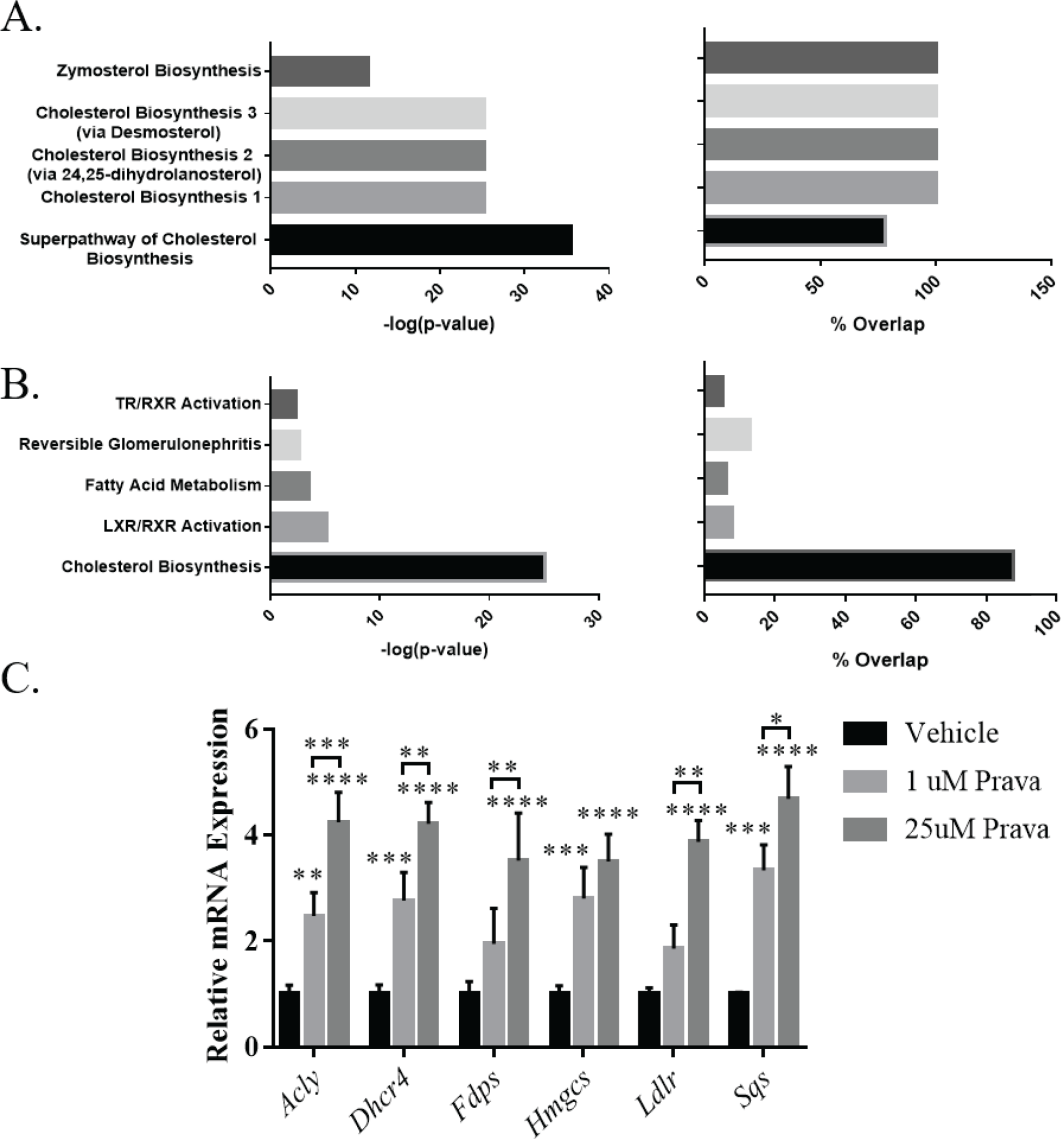
Pravastatin induces CBP gene expression in NSPCs. (A) Ingenuity pathway gene ontology analysis identified “Superpathway of Cholesterol Biosynthesis” and related pathways as the top regulated pathways in neurospheres after a 10 μM prava treatment for 24 hours.–LOG10 (p-value) and percentage overlap with identified pathways are reported (n = 6). (B) Ingenuity pathway gene ontology analysis identified “Cholesterol Biosynthesis” as the top toxicity function in neurosphere after 10 μM prava treatment for 24 hours (n = 6). (C) Average mRNA expression of cholesterol pathway and cholesterol metabolism genes in NSPCs after treatment with vehicle, 1 μM or 25 μM prava for 24 hours (n = 3–5). Expression was normalized to *GAPDH* and the vehicle treatment group for each gene. (*p<0.05, **p<0.01, ***p<0.001, ****p<0.0001 compared to controls, unless otherwise demonstrated with a horizontal bracket).

Ingenuity Pathway Analysis (IPA) was used to identify molecular pathways defined by differentially expressed genes in pravastatin versus vehicle treated NSPCs. IPA identified the Superpathway of Cholesterol Biosynthesis as the top regulated canonical pathway (p = 4.10x10^-36^) after treatment with pravastatin (Fig 4A). IPA also identified “cholesterol biosynthesis” as the top regulated toxicity pathway (p = 7.43x10^-26^) (Fig 4B). To validate that pravastatin regulates genes involved in the Superpathway of Cholesterol Biosynthesis, NSPCs were treated with high and low dose pravastatin and simvastatin for 24 hours and mRNA was analyzed by qRT-PCR. We selected several significantly regulated transcripts for qRT-PCR validation including *Acly* (*ATP Citrate Lyase*), *Dhcr24* (*24-Dehydrocholesterol Reductase*), *Hmgcs* (*HMG-CoA Synthase*), *Ldlr* (*Low Density Lipoprotein Receptor*), *Fdps* (*Farnesyl Diphosphate Synthase*), and *Sqs* (*Squalene Synthase*) and found that all were dose dependently upregulated by pravastatin (Fig 4C) as well as simvastatin (S4A Fig).

### Statin induced activation of SREBP2 in NSPCs

**Fig 5:**
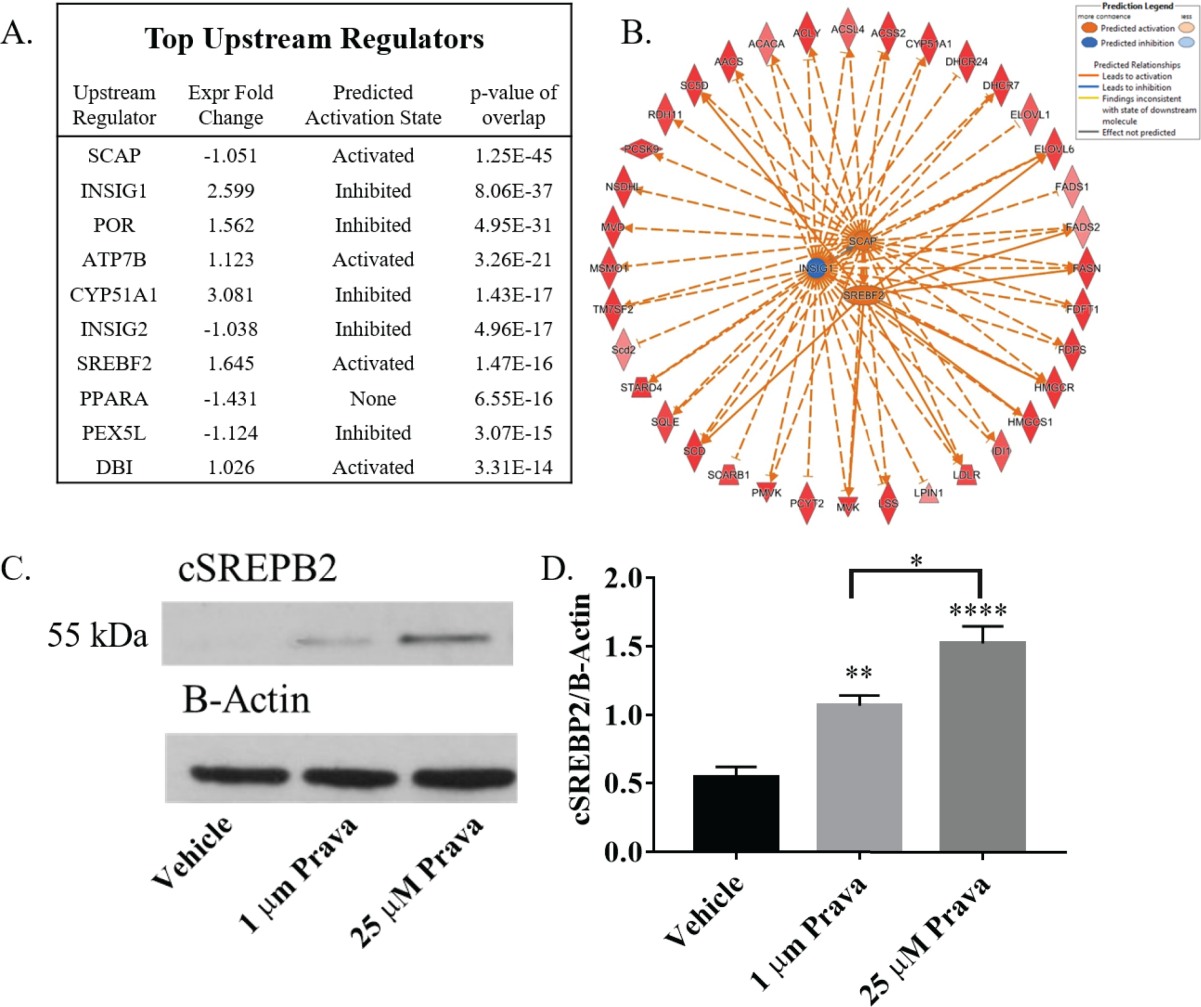
Pravastatin activates SREBP2 in NSPCs. (A) Ingenuity pathway analysis identified top upstream regulators of transcriptional change induced by 10 μM prava. (B) Network of transcriptional regulation by the SCAP-INSIG-SREBP2 complex. (C) Representative western blot of cleaved SREBP2 (cSREBP2) from NSPCs treated with vehicle, 1 μM or 25 μM prava for 24 hours. The treatment group of each band matches the column of the histogram directly below. (D) Average intensity of bands corresponding to 24 hour treatment with vehicle, 1 μM or 25 μM prava for cSREBP2 normalized to ß-Actin (n = 7). (*p<0.05, **p<0.01, ****p<0.0001 compared to controls, unless otherwise demonstrated with a horizontal bracket).

In addition to identifying molecular pathways, IPA was used to identify putative upstream regulators of differentially expressed mRNA transcripts. Of the top 10 identified upstream regulators, SCAP (p = 1.25x10^-45^), INSIG1or2 (p = 8.06x10^-37^ & p = 4.96x10^-17^, respectively), and SREBP2 (p = 1.47x10^-16^) were all found to be highly significant (Fig 5A and 5B). SCAP, INSIG, and SREBP2 form a cholesterol sensitive complex found in the endoplasmic reticulum. When cholesterol levels fall, SCAP and SERBP2 are transported to the Golgi apparatus where SREBP2 is cleaved[29]. Cleaved SREBP2 is a transcription factor that upregulates the expression of genes encoding CBP enzymes. 32 of the mRNA transcripts significantly regulated after pravastatin treatment in RNA-Seq analysis of NSPCs are included in the CBP or are important for regulation of cholesterol (Fig 5B).

To validate that the SCAP-INSIG-SREBP2 complex is activated after pravastatin treatment, NSPCs were treated with high and low dose pravastatin and simvastatin for 24 hours and cleavage of SREBP2 was analyzed by western blot. As mentioned above, SREBP2 is cleaved when cholesterol levels decrease, which triggers its nuclear localization. We observed a significant increase in the 55kDa cSREBP2 form, corresponding to cleaved SREBP2, in NSPCs treated with both low (p<0.01) and high (p<0.001) doses of pravastatin (Fig 5C and 5D). Simvastatin treated NSPCs demonstrated a significant increase in cSREBP2 in low (p<0.05) but not high dose simvastatin treatment (S4B Fig). The reason for the difference between pravastatin and simvastatin is unclear. Nonetheless, these results reveal that the SCAP-INSIG-SREBP2 complex is activated in NSPCs after statin treatment triggering an increase in the expression of CBP genes.

#### Farnesyl pyrophosphate prevents statin-induced SREBP2 cleavage and CBP gene expression

**Fig 6:**
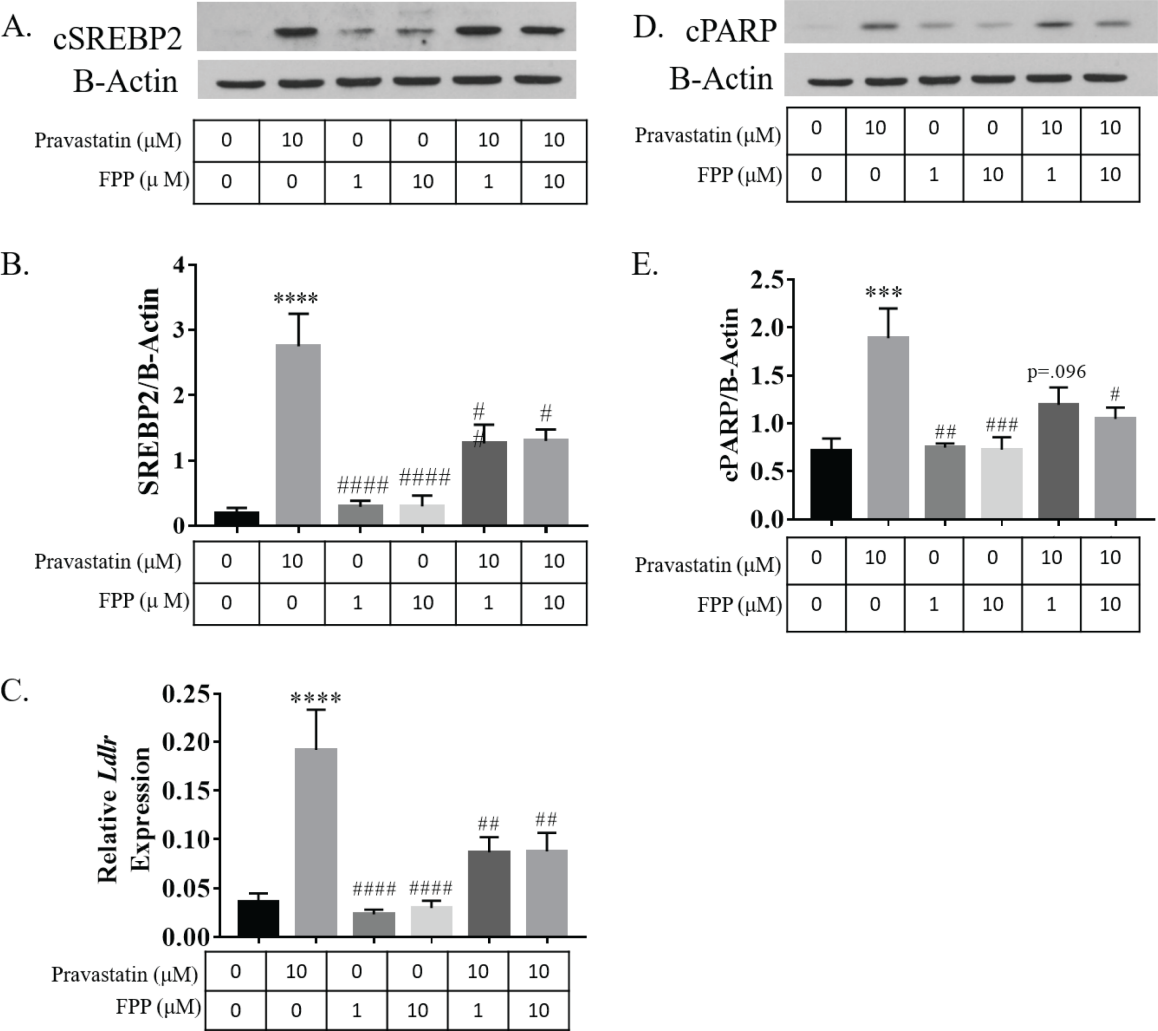
FPP prevents pravastatin dependent SREBP2 activation and induction of cell death. (A) Representative western blot of cleaved SREBP2 (cSREBP2) from NSPCs treated with vehicle, 10 μM prava, 1 μM farnesyl pyrophosphate (FPP), 10 μM FPP, 10 μM prava and 1 μM FPP, or 10 μM prava and 10 μM FPP, respectively, for 24 hours. The treatment group of each band matches the column of the histogram directly below. (B) Average intensity of cSREBP2 bands normalized to ß-actin (n = 7). (C) Average mRNA expression of *Ldlr*. Expression was normalized to *GAPDH* (n = 5). (D) Representative western blot of cleaved PARP (cPARP). The treatment group of each band matches the column of the histogram directly below. (E) Average intensity of cPARP bands normalized to ß-actin (n = 7) (***p<0.001, ****p<0.0001 compared to vehicle; #p<0.05, ##p<0.01, ###p<0.001 compared to 10 μM prava).

Statins induce SREBP2 cleavage and CBP gene expression as a response to reduced cholesterol biosynthesis triggered by HMG-CoA reductase inhibition in several cell types. To demonstrate that statin induced SREBP2 cleavage and CBP gene expression act by the same mechanism in NSPCs, we treated NSPCs with 10 μM pravastatin and either 1 μM or 10 μM of farnesyl pyrophosphate (FPP), a CBP intermediate downstream of HMG-CoA reductase and precursor to both cholesterol and isoprenoids. SREBP2 cleavage was assessed by western blot and CBP gene expression assessed by qRT-PCR of *Ldlr* expression. 10 μM pravastatin caused a significant increase in cSREBP2 (p<0.0001) compared to controls, which was prevented by co-treatment with 1 μM (p<0.01) and 10 μM (p<0.05) and FPP (Fig 6A and 6B). 10 μM pravastatin treatment also caused a significant increase in *Ldlr* expression (p<0.0001), a transcriptional target of cSREBP2, that was prevented by co-treatment with 1 μM (p<0.01) and 10 μM (p<0.01) FPP (Fig 6C). These results demonstrate that statins induce SREBP2 cleavage and CBP gene expression due in part to reduced cholesterol biosynthesis downstream of HMG-CoA reductase inhibition.

### Farnesyl pyrophosphate (FPP) prevents statin induced NSPC cell death

To test the hypothesis that statins induce NSPC cell death through inhibition of the CBP, we treated NSPCs with 10 μM pravastatin and 1 μM or 10 μM of farnesyl pyrophosphate (FPP) for 24 hours and measured cPARP, a marker of apoptosis and other forms of protease-activated cell death, by Western blot analysis. 10 μm pravastatin caused a significant increase in cPARP (p<0.001) compared to controls, which was prevented by co-treatment with 10 μM FPP, while 1 μM FPP showed a trend towards decreased PARP cleavage (Fig 6D and 6E). This demonstrates that statins cause NSPC cell death through inhibition of the CBP.

### Pravastatin and simvastatin differentially alter NSPC fate *in vitro*

**Fig 7:**
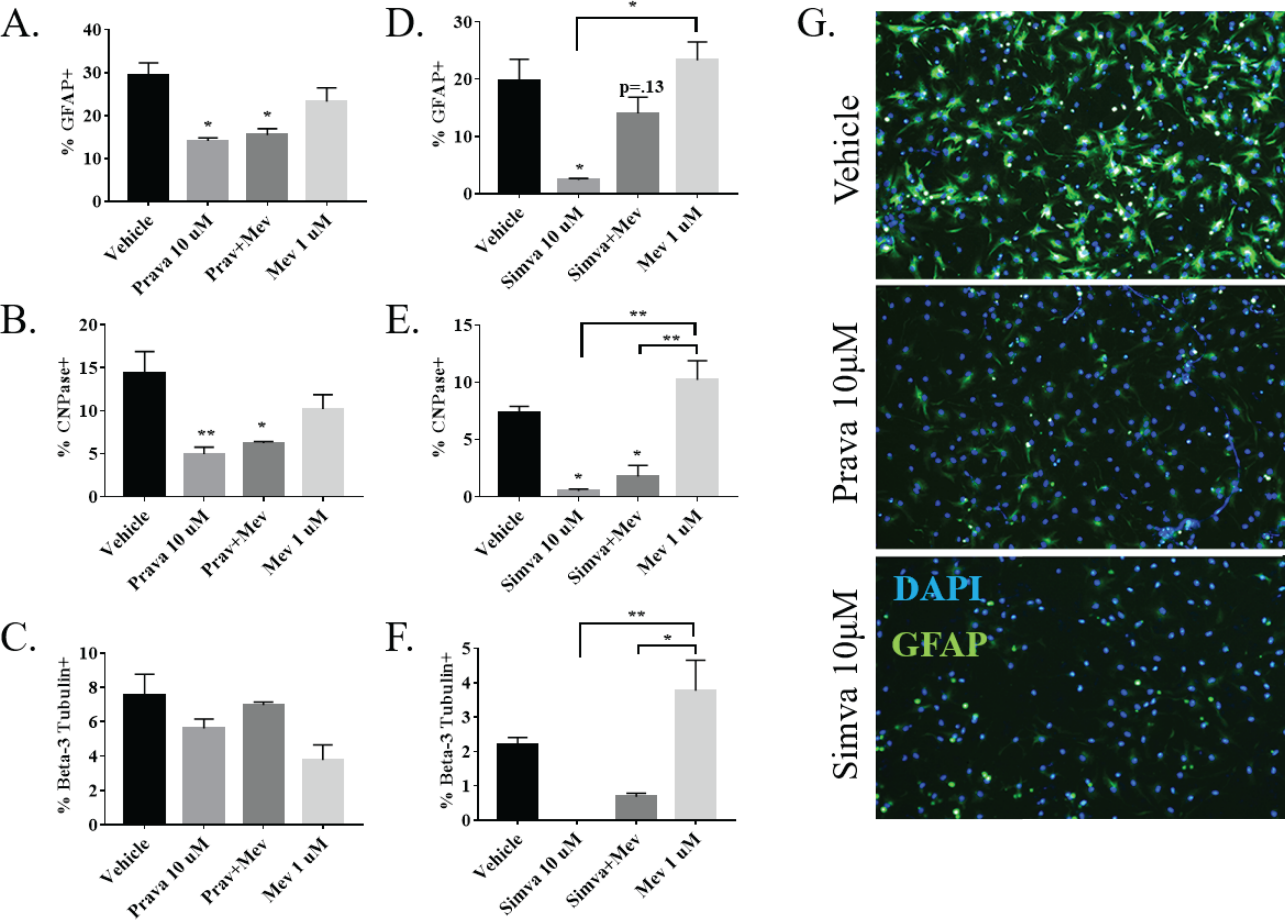
Statins differentially alter NSPC fate through a non-CBP dependent mechanism. Percentage of (A) GFAP+ glia (n = 3), (B) CNPase+ oligodendrocytes (n = 3), and (C) Beta-3 Tubulin+ neurons (n = 3) present in a population of NSPCs differentiated for three days in the presence of vehicle, 10 μM prava, 1 μM mevalonate (mev), or prava and mev. (D-F) The same experiment performed with 5μM simvastatin. (G) Representative images of differentiated cells stained with DAPI and GFAP. (*p<0.05, **p<0.01 compared to vehicle unless otherwise indicated with a bracket).

To observe direct effects of stains on NSPC differentiation and cellular fate, NSPCs were differentiated in 10 μM pravastatin or 5 μM simvastatin for three days and then subjected in immunofluorescent labeling to visualize astrocytes (GFAP+), oligodendrocytes (CNPase+), or neurons (β3-tubulin+). Pravastatin decreased the number of GFAP+ astrocytes (p<0.05), CNPase+ oligodendrocytes (p<0.01), but not neurons (Fig 7A–7C). Simvastatin also demonstrated a decrease in the percentage of glia and oligodendrocytes, but additionally decreased the percentage of neurons when compared to controls (Fig 7D–7F). This demonstrates a clear difference in the activity of pravastatin and simvastatin on differentiating NSPCs.

**Fig 8:**
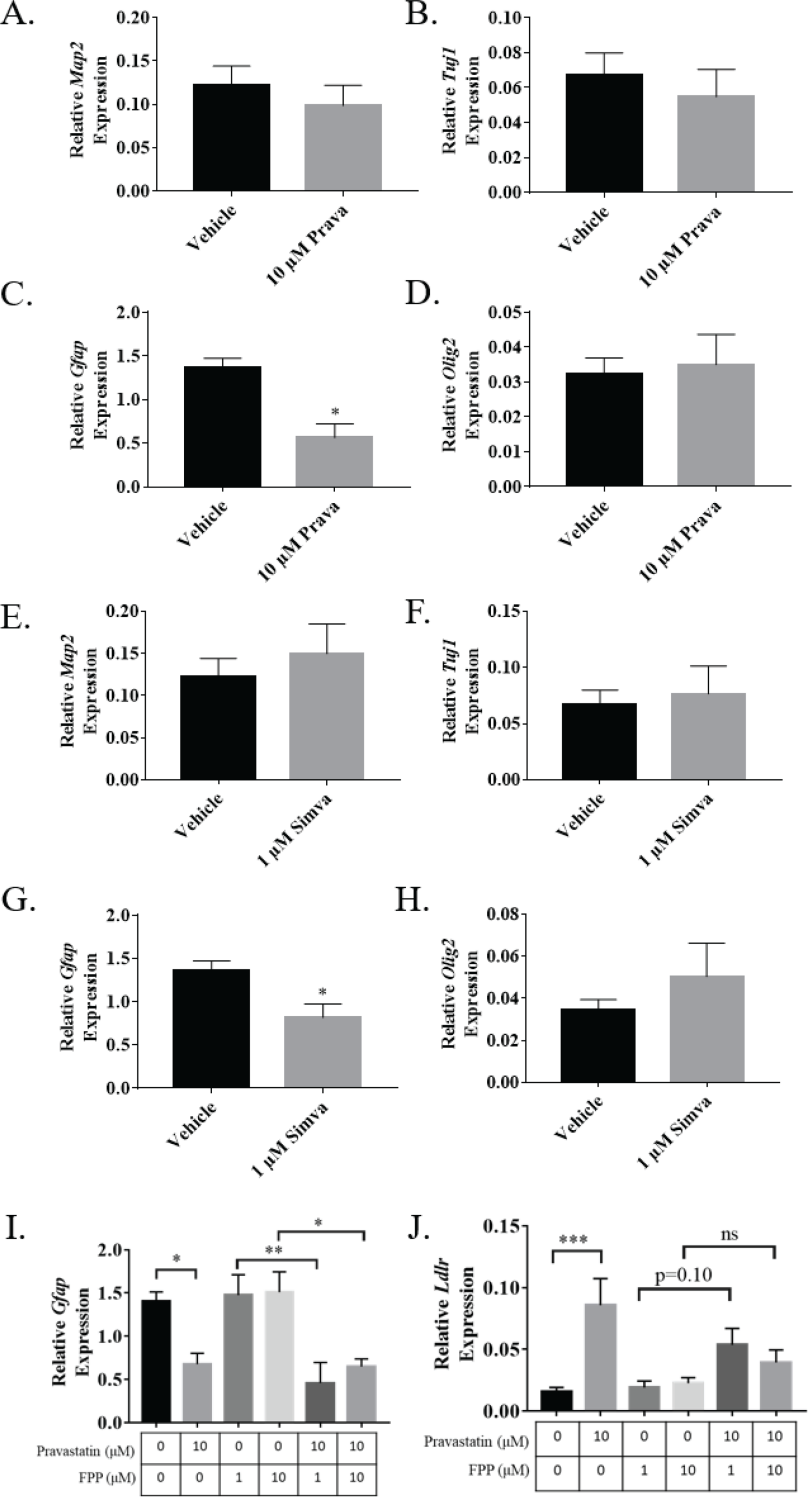
Statins alter mRNA expression in differentiating NSPCs through a non-CBP dependent mechanism. Average mRNA expression of the neuronal markers (A) *Map2* (n = 6), (B) Tuj1 (n = 6), astrocyte marker (C) GFAP (n = 6), and oligodendrocyte marker (D) Olig2 (n = 6) after four days of NSPC differentiation in 10 uM prava. Average mRNA expression of the neuronal markers (E) *Map2* (n = 6) and (F) *Tuj1* (n = 6) as well as the astrocyte marker (G) *GFAP* (n = 6) and oligodendrocyte marker (H) *Olig2* (n = 6) after four days of NSPC differentiation in 1uM simva. (I) Average mRNA expression of *GFAP* in cells after four days of differentiation in 10uM prava co-treated with 1 uM or 10 uM FPP (n = 5). (J) Average mRNA expression of *Ldlr* in cells after four days of differentiation in 10 uM prava co-treated with 1 uM or 10 uM FPP (n = 5). (*p<0.05, **p<0.01, ***p<0.001 compared to vehicle unless otherwise indicated by brackets).

In addition to examining immunofluorescence, we also examined mRNA expression in differentiating NSPCs treated with either 10μM pravastatin or 1μM simvastatin. qRT-PCR analysis of differentiated NSPCs showed no difference in mRNA expression of the neuronal markers *Tuj1* or *Map2* in response to pravastatin (Fig 8A and 8B) or simvastatin (Fig 8E and 8F) treatment. Similarly, no change was seen in the expression of the oligodendrocyte marker *Olig2* after pravastatin (Fig 8D) or simvastatin (Fig 8H) treatment. However, a significant decrease in mRNA expression of the glial marker *GFAP* occurred after treatment with 10 μM pravastatin (p<0.05) (Fig 8C) or 1 μM simvastatin (p<0.05) (Fig 8G).

#### Statins alter NSPC fate by a non-CBP dependent mechanism

To determine if statins alter NSPC fate *in vitro* through inhibition of the CBP, 1 μM mevalonate was added to differentiating NSPCs treated with 10 uM pravastatin or 5 uM simvastatin for three days. As above, differentiated NSPCs were examined for percentage of GFAP+, CNPase+, and beta-3 tubulin+ cells. Interestingly, 1μM mevalonate was unable to rescue the decrease in glia and oligodendrocytes after a 10 μM pravastatin treatment (Fig 7A and 7B). Additionally, mevalonate co-treatment was unable to rescue the decrease in glia, oligodendrocytes, or neurons after simvastatin treatment (Fig 7D–7F).

In addition to attempting rescue of the differentiation phenotype observed using immunofluorescence, we attempted rescue of the decrease in *GFAP* mRNA expression seen after 10 μM pravastatin treatment by co-treating with 1 μM or 10 μM FPP. The decrease in *GFAP* mRNA expression was not prevented by either co-treatment with 1 μM or 10 μM FPP compared to their respective controls (Fig 8I). To rule out the possibility that neither dose of FPP was sufficient to prevent transcriptional changes in the differentiation assay, expression of *Ldlr* was quantified in differentiated cells. Like proliferating NSPCs, differentiated cells treated with 10 μM pravastatin demonstrated a significant increase in *Ldlr* expression (p<0.001) and when co-treated with 1 μM (p = .10) or 10 μM FPP demonstrate a return to baseline levels of expression (Fig 8J). These results are consistent with immunofluorescence data and suggest that statins alter NSPC fate through a non-CBP dependent mechanism.

## Discussion

Statins are currently being evaluated for the prevention of preeclampsia in high-risk pregnant women [9] despite their initial designation as a category X drug. There is a clear need to develop pharmacotherapies for preeclampsia, as current treatment options are extremely limited and culminate in emergency delivery, regardless of gestational age [30]. We therefore evaluated the effect of two statins with distinct pharmacokinetic properties on primary cultures of NSPCs derived from embryonic mouse cerebral cortex. We chose to isolate NSPCs at embryonic day 14.5 because it is a period of mouse brain development which closely reflects human brain development in the third trimester [31] where statin treatment might be therapeutically beneficial in the prevention of preeclampsia.

We discovered that pravastatin, a hydrophilic statin, and simvastatin, a lipophilic statin, both dose dependently inhibit the expansion of NSPC populations by inducing cell death during in NSPCs in G1. We also observed a robust increase in CBP gene expression after treatment with pravastatin and simvastatin suggesting that NSPCs respond to statin induced inhibition of HMG-CoA reductase by increasing enzymes involved in the CBP. The increase in CBP gene expression is likely mediated by activation of the SCAP-INSIG-SREBP2 complex, which regulates cholesterol homeostasis in other tissues [29]. Despite dose dependent transcriptional upregulation of CBP genes, statins induce NSPC apoptosis suggesting that this response is not sufficient to overcome a cytotoxic response, particularly at high statin concentrations. When considering the use of statins prenatally, it will be important to take the dose dependent increase in apoptosis into consideration.

To minimize the dose seen by the developing brain, solubility of statins is important to consider, as lipophilic molecules are more easily able to cross biological barriers. Pravastatin is hydrophilic and has a decreased potential to cross the blood-brain barrier and placental barrier compared to lipophilic statins, including simvastatin; therefore, limiting the accumulation in the developing brain [32,33]. Another important clinical consideration for use of this drug would include the evaluation of patients for who have relevant genetic variations in drug transporters and enzymes, which aid in clearance and metabolism of statins. Both drug transporters in the ATP-binding cassette (ABC) transporters and cytochrome P450 superfamilies have been implicated in clinical responses to statins [34,35]. Of interest is the ABCB1 drug transporter, which is expressed in both the blood-brain barrier [36] and placenta [37] to limit xenobiotic exposures. Therefore, future pre-clinical studies that would extend our studies of fetal responses to statins could be designed to examine the impact of placental and fetal cytochrome P450 enzymes and drug transporters on neurodevelopment.

We demonstrated that NSPC cell death is due to inhibition of the CBP since PARP cleavage induced by statins in NSPCs is overcome by treatment with FPP. Products of the CBP including cholesterol and isoprenoids are essential in both early and late stages of brain development. Cholesterol plays many important roles in NSPCs including lipid raft formation, steroid hormone biosynthesis, and fat soluble vitamin synthesis including retinoic acid [17]. Because the cholesterol metabolism of the brain is isolated from the rest of the body [38], statins deserve special consideration during development since they inhibit *de novo* synthesis of cholesterol. Isoprenoids include FPP, which is the direct precursor to both squalene and GGPP, and GGPP. These small molecules are involved in protein prenylation, which facilitates tethering to lipid membranes. Prenylated proteins including lamin B and several GTP-binding regulatory proteins (G-proteins) play essential roles in neurodevelopment [39,40]. In fact, statins inhibit primitive streak formation by inhibiting the synthesis of FPP, not cholesterol in mouse embryonic stem cells [39]. In addition, there is a wealth of research on the role that prenylated G-proteins, such as Rho family GTPases, play in neurodevelopment, survival and degeneration [40]. In addition, the observation that statins modestly increase autophagy in NSPCs opens the door to future studies that could elucidate the impact of autophagy on NSPC survival and proliferation.

In NSPCs derived from the hippocampus adult mice, simvastatin causes preferential differentiation into neurons *in vitro* [20]. The same report also demonstrated a trend towards fewer astrocytes. Here, we demonstrate that pravastatin does not change the ability of NSPCs to differentiate in neurons, but that simvastatin surprisingly decreases the differentiation of NSPCs derived from the cortex of embryonic mice into neurons. Interestingly we also observed that that both pravastatin and simvastatin cause a significant decrease in the number of GFAP+ glia and CNPase+ oligodendrocytes. The differences observed could be due to differences in the age of the animals or the site of tissue collection. Regardless, both pravastatin and simvastatin influence the cell fate of embryonic cortical NSPCs, via mechanisms that will require more thorough investigation. Interestingly, altered cell fate after treatment with statins does not appear to be due to inhibition of the CBP since the addition of FPP was not sufficient to rescue the decrease in GFAP+ mRNA, and mevalonate was insufficient to rescue the changes in NSPC fate. This could be due to off-target effects of statins, such as activation of PPAR receptors [8] or another undiscovered mechanism.

We have demonstrated that pravastatin and simvastatin prevent NSPC expansion *in vitro* by inducing dose-dependent NSPC cell death, likely during G1 of the cell cycle. In response to statin treatment, NSPCs exhibit a dose-dependent increase in CBP gene transcription through activation of SREBP2, which can be prevented by co-treatment with FPP. Finally, we demonstrated that pravastatin and simvastatin each uniquely alter NSPC cell fate via a mechanism that does not appear to be related to altered cholesterol biosynthesis. The known differences in ability to cross the placental and blood-brain barrier, in addition to dose dependent nature of NSPCs to statin and unique effects on differentiation demonstrated in this paper suggests that treatment paradigms may be attainable that provide benefit to pregnant women with what is often an intractable condition but exert limited if any adverse neurodevelopmental effects in the fetus.

## Supporting information

S1 Fig Simvastatin decreases NSPC expansion *in vitro*. **(**A) Quantification was performed on images taken in triplicate using image J software on days 0, 1, 3 and 5 after treatment with vehicle, 0.1uM or 5uM simvastatin (simva) (n = 5). (B) After imaging, NSPCs were collected for automated cell counting in duplicate (n = 5).
(EPS)

S2 Fig Simvastatin induces NSPC apoptosis and cell cycle dysregulation. (A) Diva software was used to count the number of NSPCs, which were identified as apoptotic, in G0/G1 or in S/G2/M phase. The histogram represents the mean fraction of NSPCs, which are apoptotic, in G1, or in S/G2/M of the cell cycle after treatment with vehicle, 0.1 uM, or 5 uM simva (n = 5). (B) Average intensity of bands corresponding to 24 hour treatment with vehicle, 0.1 uM or 5 uM simva for cleaved PARP normalized to ß-actin (n = 5). (C) Average expression of *Ccnd1* mRNA as assessed by qRT-PCR (n = 5). (D) Average intensity of bands corresponding to 24 hour treatment with vehicle, 0.1 uM or 5 uM simva for cyclin D1 normalized to ß-actin (n = 5). (*p<0.05, **p<0.01, ***p<0.001 compared to controls, unless otherwise demonstrated with a horizontal bracket).
(EPS)

S3 Fig Pravastatin increases autophagy. (A) Representative western blot of LC3B-I (top) and II (bottom) with α-tubulin control. (B) Quantification of LC3B-II/(LC3B-I+LC3B-II) protein ratio. (***, p<0.01)
(EPS)

S4 Fig Simvastatin induces compensatory CBP gene expression and activation of SERBP2. (A) Average mRNA expression of cholesterol pathway and cholesterol metabolism genes in NSPCs after treatment with vehicle, 0.1 uM or 5 uM simva for 24 hours. Expression was normalized to GAPDH and the vehicle treatment group for each gene (n = 3–5). (B) Average intensity and SEM of bands corresponding to 24 hour treatment with vehicle, 0.1 uM or 5 uM simva for cleaved SREBP2 (cSREBP2) normalized to ß-actin (n = 7) (*p<0.05). (*p<0.05, **p<0.01, ***p<0.001, ****p<0.0001 compared to controls, unless otherwise demonstrated with a horizontal bracket).
(EPS)

S1 Table Top 25 pravastatin regulated genes. Genes in NSPC after treatment with prava ordered by p-value (n = 6).
(EPS)
